# Finding *ikigai*: How robots can support meaning in later life

**DOI:** 10.3389/frobt.2022.1011327

**Published:** 2022-10-24

**Authors:** Natasha Randall, Swapna Joshi, Waki Kamino, Long-Jing Hsu, Abhijeet Agnihotri, Grace Li, Donald Williamson, Kate Tsui, Selma Šabanović

**Affiliations:** ^1^ R-House Lab, Indiana University, Luddy School of Informatics, Computing, and Engineering, Bloomington, IN, United States; ^2^ Robotics User Experience and Industrial Design, Toyota Research Institute, Cambridge, MA, United States; ^3^ ASPIRE Research Group, Indiana University, Luddy School of Informatics, Computing, and Engineering, Bloomington, IN, United States

**Keywords:** home robots, social robots, human-robot interaction, meaning in life, eudaimonic well-being, happiness, older adults, *ikigai* (sense of life worth living)

## Abstract

Previous research in human-robot interaction has explored using robots to increase objective and hedonic aspects of well-being and quality of life, but there is no literature on how robots might be used to support eudaimonic aspects of well-being (such as meaning in life). A sense of meaning has been shown to positively affect health and longevity. We frame our study around the Japanese concept of *ikigai*, which is widely used with Japanese older adults to enhance their everyday lives, and is closely related to the concept of eudaimonic well-being (EWB) known in Western countries. Using a mixed-methods and exploratory approach, including interviews with 17 older adults and the collection of 100 survey responses, we explored how older adults in the US experience a sense of meaning, and if and how a social robot could be used to help foster this sense. We find that meaning for older adults is often obtained by helping others, through family connections, and/or through activities of daily life, and that sources of meaning often differ based on the older adults’ living situation. Assessing how meaning compares to happiness and social connection, we highlight general similarities and differences, and also find that living situation influences older adults’ sources of happiness, desire for social connection, and barriers to well-being, in addition to companionship and happiness having a weaker correlation with meaning for those who live alone than for those who live with others. Additionally, we evaluated initial perceptions of a social robot (QT) meant to enhance *ikigai* and overall well-being, finding mostly positive perceptions, though those who live alone also reported being less willing to adopt a social robot into their homes. Using both data collected on older adults’ meaning and the potential use of QT to support meaning, we make several design recommendations with regards to using robots to enhance *ikigai*, such as by prompting daily reflecting, enhancing family bonds, and suggesting new experiences and volunteer opportunities.

## 1 Introduction

There is consensus among most researchers that well-being falls into two broad categories—hedonia and eudaimonia—and that it is necessary to assess both aspects when measuring well-being (Ryan and Deci, 2001). Increasing hedonia (e.g., high positive affect, low negative affect, and life satisfaction), has been the focus of much human-robot interaction (HRI) and social robotics research with older adults (OAs). However, work on whether and how robots can help older adults maintain and increase eudaimonic aspects of well-being (e.g., meaning, vitality, self-connection, personal growth, accomplishment, etc.,) has been missing from the literature. Since *meaning* captures much of the variance in eudaimonic well-being (EWB) and is often used as its proxy (Huta, 2017), in this paper, we focus mainly on understanding OAs meaning and purpose in life and exploring how a social robot might be designed to support it.

While the *meaning of life* may seem like an elusive concept, a sense of purpose and meaning is essential to health throughout one’s life span. For OAs, recognition that one has a purpose in life can have a positive effect on longevity and overall well-being, which correlates with fewer chronic health conditions, less disability (Krause, 2009), reduced mortality (Musich et al., 2018), and better health behavior outcomes (Kim et al., 2020). At the same time, changing life circumstances that accompany aging—retirement, children leaving the home, health issues—can challenge people’s established sense of purpose. OAs, therefore, may need to re-establish their sense of purpose and meaning as their lives and abilities change. To address these concerns, therapists have developed ways to increase OAs’ sense of purpose and meaning (Chippendale and Boltz, 2015), and various organizations have implemented interventions to improve OAs’ sense of purpose. These include “ikigai centers” emphasizing socialization and skill development in Japan, and opportunities for volunteering and peer support in the United States (Owen et al., 2021).

Our work investigates how robots may be designed to support OAs sense of purpose and meaning. This research can expand the preventative and therapeutic functions of socially assistive robots, and enhance existing approaches to maintaining well-being in later life. We base our studies on the Japanese concept of *ikigai*, which combines two Japanese characters: “*iki*,” or life, and “*gai*,” or value/worth. In English, ikigai most closely translates as a person’s meaning in life or reason for living (Mathews, 1996b; Mitsuhashi, 2018), and has been found to be conceptually similar to EWB (Kotera et al., 2021). Ikigai encompasses social participation, though with a focus on doing something *for* others, instead of simply *with* others (Kumano, 2018). EWB (and by extention, *ikigai*) has also been highly correlated with happiness, with long-term studies showing that it increases happiness (hedonia) over the long-term, while the reverse does not appear to occur (Ring et al., 2007). We use the concept of ikigai as the basis of our work due to its wide and established use in supporting older adults in Japan along with its rich conceptual framework.

In this paper, we explore 1) how OAs define and experience a sense of meaning and purpose, and 2) how robots might help OAs maintain and expand their sense of meaning in life–their *ikigai*. We also consider how the design of robots to support OAs’ sense of meaning and purpose may align with or differ from robots more commonly used to support well-being in HRI studies, which emphasize increasing happiness and social connectedness. We build on prior work suggesting that robots for OAs should incorporate an understanding of the positive aspects of aging and seek to help OAs to maintain those experiences (Lee et al., 2016; Cahill et al., 2018).

This exploratory research employs surveys and interviews with OAs to understand the possibilities for using robots to support meaning-making and related activities. We include two groups of OAs in our study—those living at home, and those in an assisted living facility—to understand diverse experiences of OAs and to frame our design insights accordingly. Our studies seek to incorporate the perceptions, needs, and preferences of OAs from the first steps of the design process, thereby avoiding the pitfalls of designs that view aging mainly as a disability (Lee et al., 2016).

## 2 Background

### 2.1 Aspects of well-being

Well-being is divided into two branches: hedonic and eudaimonic (Huta, 2017). Hedonia, a state characteristic more commonly known as happiness, is commonly the target of studies in HRI, with use of measures such as the UCLA Loneliness Scale and the Positive and Negative Affect Scale (PANAS), among others. Positive affect, negative affect, and life satisfaction are the three common construct measures of happiness (Martela and Sheldon, 2019).

Eudaimonic well-being (EWB), on the other hand, has struggled to reach a consensus as to its definition in literature (Martela and Sheldon, 2019), only being clearly defined by what it is not (i.e., not mere affect, pleasure, or happiness) (Proctor and Tweed, 2016). Therefore, it encompasses many important aspects of one’s experiences, including meaning in life, vitality, spiritual transcendence, accomplishment, engagement, and self-acceptance. However, most scholars agree that if a single construct is to be associated with EWB, it is meaning. In fact, meaning has been found to capture 70% of the variance in EWB (Huta, 2017), and it is often used as a proxy for EWB (Huta, 2017).

Hedonic well-being is associated with a number of positive health outcomes, but so too is EWB. EWB is associated with higher degrees of physical health (Roepke et al., 2014), mental health (Heisel and Flett, 2004; Mascaro and Rosen, 2005), and social functioning (Stillman et al., 2011; Stavrova and Luhmann, 2016). People who have high degrees of both hedonia and eudaimonia experience higher levels of well-being and mental health than those who pursue only one type (Keyes et al., 2002; Huta and Ryan, 2010; Anic and Tončić, 2013). Even though hedonic well-being is associated with higher levels of well-being than EWB in the short-term (Huta, 2017), EWB has been shown to be more important than hedonia (happiness) for *long-term well-being* (Steger et al., 2008; Huta and Ryan, 2010), potentially because EWB has been found to influence happiness, while the inverse is not true (Joshanloo, 2018).

Quality of life (QOL) research, which is related to well-being research in many ways but often emphasizes the relationship between objective and subjective measures of QOL, has also recognized the importance of incorporating eudaimonic aspects into its measures. This is reflected in the World Health Organization’s (WHO’s) most recent definition of QOL, which now includes purpose in life. However, updates to QOL scales (i.e., WHOQOL SRPB, WHOQOL SRPB BREF) (World Health Organization and others, 2002; Skevington et al., 2013), have yet to be reflected in HRI research. Research has also shown that the related concept of *ikigai* (detailed below) is a unique construct that should be added to commonly used QOL measures (Demura et al., 2005). This means that older and consistently used versions of QOL scales have not reflected, or have not adequately reflected, a eudaimonic component.

Though the importance of assessing and positively addressing EWB is clear, research in HRI and social robotics has yet to explore how robots might be useful for this purpose.

### 2.2 *Ikigai* and related concepts in studies with OAs

The Japanese term *ikigai* broadly refers to that which makes one’s life worth living (Mathews, 1996a). It can also encompass the meaning of life (Hasegawa et al., 2001), well-being (Kumano, 2018), self-realization (Tsukasa, 1989), or simply, the joy a person finds in living day-to-day (Mitsuhashi, 2018). Researchers distinguish between two constituent aspects of ikigai: *“ikigai-kan”* which is the **feeling of ikigai**, and *“ikigai tai-sho”* which is the object or the **source of ikigai** (Kamiya, 1966).

Today, Japanese policy makers and researchers widely accept that ikigai is essential for OAs to lead fulfilling and independent lives (Uehara, 2005). Municipal governments and councils of social welfare, among others, organize various activities to formally support OAs’ ikigai in (*e.g.,* “ikigai centers” and “iki-iki salons”). In the past few decades, researchers in Japan have developed three scales and conceptual models specifically for measuring ikigai: the K-1 scale (Kondo and Kamada, 2003), the constituent-based model (Hasegawa et al., 2001), and the Ikigai-9 scale (Imai et al., 2012). The ikigai-9 has been validated in the UK (Fido et al., 2020).

Researchers have also investigated ikigai in the US (Mathews, 1996a; Fujita-Sano, 2014). These studies suggest that, while no exact equivalent to ikigai exists in the English vocabulary, there is a parallel sense of ikigai as *“what one most deeply lives for”* in the US as well as Japan (Mathews, 1996a). It has also been found to be mostly synonymous with the concept of EWB (Kotera et al., 2021). Ikigai in both countries includes individual as well as social components, such as personal skill-building and contributing to the community (Mathews, 1996a; Fujita-Sano, 2014). In fact, it has been described as having **three “levels”—1st person (personal), 2nd person (interpersonal), and 3rd person (community)**. Ikigai sources of meaning may occur at any one of these levels, though is ideally experienced at all three levels (e.g., personal hobbies, family, and volunteering) (General Incorporated Foundation Health and Purpose of Life Development Foundation, 2019).

Along with studying how OAs in the US define and experience meaning in life—their *ikigai*—research on happiness, quality of life, and social participation and connectedness all go beyond the physical determinants of health to incorporate positive psychological and social experiences that provide measurable benefits to OAs. Identifying how robots can support these important subjective experiences contributes to the expansion of possible health applications of HRI.

### 2.3 Social robots to support OAs’s everyday well-being

While using social robots with older adults may present certain ethical issues, such as loss of control, possible reduction in human contact, and declining privacy (Pedersen et al., 2018; Van Maris et al., 2020), older adults have often indicated a desire to interact with them and received benefits from their use. For instance, research shows that OAs also desire companionship with robots in activities such as dining, resting, doing housework, and entertaining (Chen et al., 2019). Social robots can also be designed to help OAs improve their levels of social participation by reminding and suggesting how they can connect with others in local or remote activities (Cahill et al., 2018; Obayashi et al., 2020). For example, ElliQ, a minimally anthropomorphic robot with a moving head and accompanying tablet, uses speech and nonverbal cues to connect OAs who have dementia to their family and friends; it also comforts, entertains, reminds, and suggests activities for them (Deutsch et al., 2019). Paro, a zoomorphic seal robot, has been used in single family homes and nursing homes for both social companionship and social facilitation (Chang et al., 2013; Randall et al., 2019). This ability of some social robots to support social facilitation is interesting, as it shows that robots can be designed or used in a way that alleviates certain ethical concerns (reduction of human contact, in this case). The Mabu robot, a healthcare robot with some anthropomorphic features, uses verbal communication to gather health-related data from its users and provide behavioral reminders and suggestions personalized to individuals (Clabaugh and Matarić, 2018). Along with monitoring, social robots are often used to reinforce specific behaviors that can improve well-being (Scoglio et al., 2019), such as physical exercises (Fasola and Matarić, 2013; Görer et al., 2017; Lotfi et al., 2018) and better medication management (Smarr et al., 2012; Su et al., 2021).

Together, these studies suggest that well-being support is a promising application domain for social robots to benefit OAs. Our research aims to add to this research by exploring how robots might support OAs in being more personally active and societally engaged, in addition to being connected socially to their existing network, in ways that are meaningful to them.

## 3 Materials and methods

**FIGURE 1:**
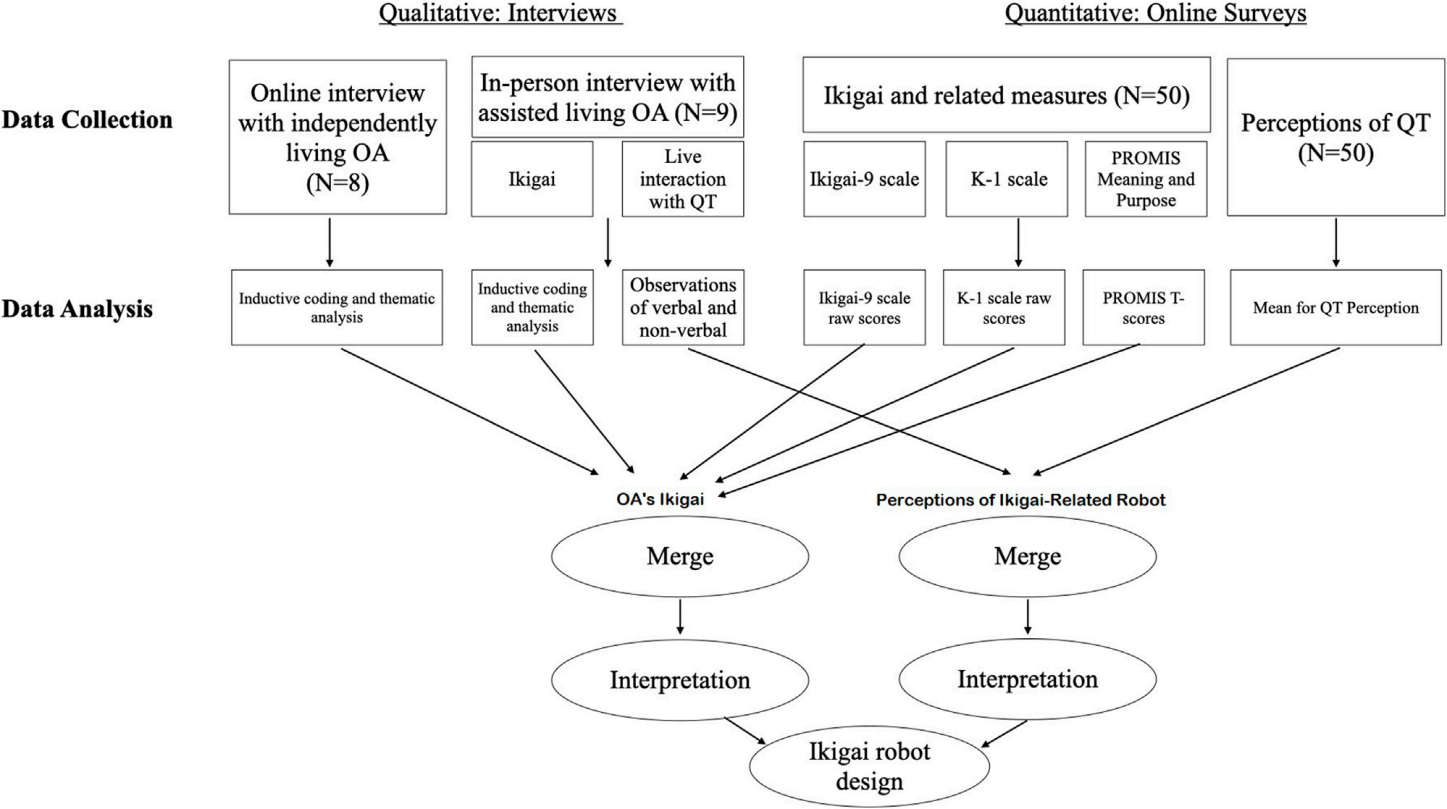
Procedural diagram of the mixed-methods study design.

**FIGURE 2:**
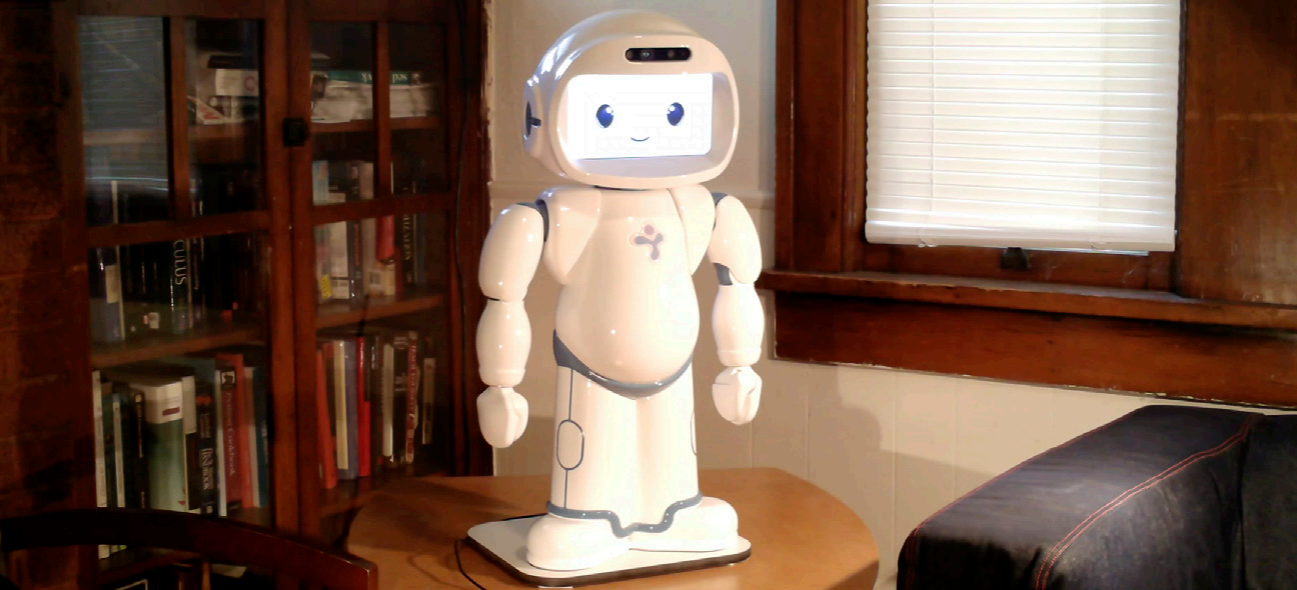
QT as filmed in R-House Lab at Indiana University.

Our mixed methods design (Figure 1) included online surveys (one on *ikigai* and related measures, and one on perceptions of the QT robot^1^), and interviews with independently living (home, independent senior housing) and assisted-living OAs, to include diverse OA perspectives. We showed participants LuxAI’s QT (Figure 2), a programmable humanoid robot. Outfitted with microphones, speakers, and 3D cameras, QT provides a wide array of communication and interaction capabilities for HRI design. Survey and interview participants recruited via Amazon Mechanical Turk (M-Turk) were compensated with a $10 payment and a $20 Amazon gift card, respectively. The AL facility, interested in scientific contribution and opportunities for their residents to socialize and participate in research activities, opted out of participant compensation. The research was approved by Indiana University’s research ethics board.

### 3.1 Participants and study setting

Participants were at least 65 years old and residents of the United States. Demographic details for participants in the various components of the study are given in Table 1.

**TABLE 1:** Participants demographics.

	Online survey (Ikigai)	Online survey (Robot Perception)	Online Interviews (M-Turk)	In-person Interviews (AL)
	(*N* = 50)	(*N* = 50)	(*N* = 8)	(*N* = 9)
Gender	24 M/26 F	29 M/21 F	3 M/5 F	2 M/7 F
Age	*μ* = 68.4	*μ* = 68.0	*μ* = 68.5	*μ* = 83.5
	*σ* = 3.5	*σ* = 3.1	*σ* = 4.1	*σ* = 6.0

Online surveys were deployed on M-Turk to collect data from more geographically diverse OAs (Berinsky et al., 2012). M-Turk samples have often been shown to be more representation than census-based panels in certain areas and generalize well to certain health outcomes (Mullinix et al., 2015; Redmiles et al., 2019; Merz et al., 2020). Once completed, survey responses were reviewed; responses were excluded if participants gave nonsensical answers to open-ended questions or if they did not meet the age requirement stated at the beginning of the survey. 50 completed responses to each survey were collected; about half of these respondents (24) completed both surveys. Nearly all participants reported moderate or frequent use of data communication technologies (e.g., cell phones, internet), though few had used robots in the past, with the exception of Roomba.

Eight OAs were also recruited from M-Turk for the online interviews, conducted through Google Meet. We used purposive sampling to recruit them, in order to help explain survey findings. We asked about their sense of happiness, meaning in life, and social support. These questions were meant to help us interpret the quantitative results from the initial surveys.

For the in-person interviews, we recruited nine OAs from a local Assisted Living (AL) and memory care facility, which provides in-house nursing and companion care support for activities of daily living (ADL) and scheduled social and recreational activities for its residents and day visitors in a community atmosphere. Seven OAs were residents, and two were day visitors. Participants were not screened for cognitive decline, but staff assisted with recruitment of high functioning OAs. Though participants had previous exposure to a pet robot (JoyForAll), this was their first experience with an anthropomorphic robot. Interviews were held in a private room at the AL facility.

### 3.2 QT introduction to participants

Online M-Turk participants were introduced to the QT robot by a video, while in-person AL participants interacted with the robot in a live demonstration^2^. QT was chosen as it is a commercial robot with a rich SDK, making it more robust for in-home use and widely adaptable by researchers.

The capabilities and activities presented in the **video of QT** were based on literature related to ikigai and well-being. The video showed: social engagement prompting (e.g., QT: *“Maybe you should call Mary later to share what happened?”*), storytelling prompting, emotion mirroring (e.g., QT: *“I’m feeling low today. Exercise always cheers me up. How about doing some exercises together?”*), exercise, skill and cognitive development, game play, personalization through user programming, and reflection prompting (e.g., QT: *“You’ve done well today. Before you go to bed, reflect on one thing you were proud of today and one thing you could do tomorrow to help increase your sense of meaning.”*). These interactions exemplified how QT might help improve OAs’ source and sense of ikigai through activities and self-awareness. We also showed the robot being proactive (initiating conversations and offering activity suggestions). The video mentioned the ability of QT to make suggestions for increasing meaning-making activities or social interaction at personal, interpersonal, and community levels. The video was recorded in a naturalistic, home-like environment to show QT in its potential context of use. Only the robot was shown. The narrator both described the robot, and acted as its interlocutor. The video was 3 min and 55 s long.

The robot capabilities displayed in **live interaction with QT** were consistent with the video presentation. The robot proactively initiated conversations based on topics and offered activity suggestions parallel to those in the video. QT’s interactions with individual participants were chosen by the researcher (“wizard”) from a pre-selected set. AL participants interacted with QT for approximately 5 min each, except for one participant who left early due to a scheduling conflict.

### 3.3 Online survey protocols

The online surveys for M-Turk participants collected rating scale data and written responses to open-ended questions. We created two different surveys using Qualtrics—one to measure subjective happiness, meaning, and social support (*Ikigai* Survey), and the other to determine perceptions towards QT as based on a video (QT Perception Survey). Two separate surveys were used to keep completion time manageable, as data quality has been show to decrease with survey length (Galesic and Bosnjak, 2009). These were simultaneously deployed on M-Turk.

#### 3.3.1 Ikigai survey

The participant self perception survey included validated scales measuring *ikigai* and related concepts. The included *K-1 scale* (Kondo and Kamada, 2003) is widely used with OAs in Japan, while the *ikigai-9 scale* (Fido et al., 2020) is frequently used in surveys conducted by the municipal/regional governments in Japan. We included four *PROMIS subscales* selected from the National Institute of Health’s Patient-Reported Outcomes Measurement Information System (PROMIS) to measure meaning and purpose, positive affect, companionship, and emotional support (Hahn et al., 2010; Broderick et al., 2013; Hanmer, 2021). We used these scales to empirically explore the relationship between PROMIS measures developed in the US and ikigai measures developed in Japan.

We included a technology familiarity scale (Ezer et al., 2009), and questions on social interaction frequency, demographics, and health. We asked open-ended questions about activities that bring participants a sense of meaning or happiness, and whether the COVID-19 pandemic influenced their answers. Participants completed this survey in around 25 min.

#### 3.3.2 QT perception survey

In the online survey, participants watched the video demonstrating QT’s general well-being and *ikigai*-related features and interaction capabilities before answering the QT perception questions. These included the *Almere scale* (Heerink et al., 2010), developed to measure OAs’ acceptance of social robots, the same *technology familiarity scale* and health and demographic questions used in the ikigai survey above, and questions on feelings toward home use of the robot as well as comfort with discussing experiences, memories, strengths, and goals with it. It also included questions regarding feelings on the robot’s intrusiveness and proactivity, and open-ended questions on daily activities participants might do with the robot. Participants completed this survey in approximately 15 min.

### 3.4 Interview protocols

We performed online interviews with M-Turk participants to get a more in depth understanding of their sense of purpose and meaning that could help us interpret survey results. Our interviews with AL participants included verbally going over the survey questions used with online participants, as well as open-ended questions to understand participants’ sense of purpose in their own words. The open-ended questions in both online and in person interviews were based on those suggested by Mitsuhashi (2018). All interviews were video-recorded.

#### 3.4.1 Online interview protocol

Semi-structured interviews were conducted with participants to identify individuals’ sense and sources of happiness, meaning, and social support. Participants were also asked about their willingness to have any robotic technology in their homes to help them in the areas mentioned above. Interviews averaged 30 min in length.

#### 3.4.2 In-person interview protocol

With in-person participants, we first went over the open-ended questions on *ikigai*-related experiences and activities (Mitsuhashi, 2018). We then verbally administered the contents of the *Ikigai* Survey, allowing appropriate response time and opportunity to provide more open-ended comments on the questions. Following the *ikigai*-related questions, participants took part in a live interactive demonstration with the QT which showcased ikigai and well-being related behaviors, as described above. Then, participants answered questions about their perceptions of QT and its potential use in their home, based on the QT Perception Survey provided to online participants. We did not test whether the robot improved their sense of meaning in life, as this is relatively constant over time (i.e., would require testing after longer-term interaction). We only assessed willingness to use the robot for such a purpose. Interviews took approximately 1.5 h and were conducted in a single session.

### 3.5 Data analysis

For the surveys, we analyzed all four *PROMIS subscales* by calculating raw scores then converting these scores to T-scores according to (National Institutes of Health, 2014; National Institutes of Health, 2017; National Institutes of Health, 2018a; National Institutes of Health, 2018b). These standardized scores are based on the US general population. The average has been set to a score of 50, and a 10-point derivation is equivalent to one standard deviation difference. We analyzed the *ikigai-9 scale* by calculating raw scores. For the *K-1 scale*, raw scores were calculated and level of *ikigai* was determined per the guidelines outlined in (Kondo, 2007). Written answers to open-ended survey questions on happiness and meaning, and robot use, were coded using inductive coding; 19 codes resulted for the former and 13 for the latter. Furthermore, we explored whether there were notable differences in survey responses based on gender and whether participants lived alone.

Interview data of assisted living (*N* = 9) and M-Turk (*N* = 8) participants^3^ were transcribed using Otter.ai, then exported to Dedoose for inductive coding. Three authors, all with a background in HRI, were involved in thematic analysis of interview data, based on the ‘coding reliability approach’ (Braun and Clarke, 2021; Finlay, 2021). All coders individually coded and generated themes for 20% of the data. After several rounds of discussions leading to a coding scheme, 196 codes were created and used to code the data. This resulted in 37 top-level categories, including barriers to well-being, sources of meaning, sources of happiness, activities performed, feelings expressed, means of connecting with others, and sources of contribution. Approximately 10% of the data was coded to measure inter-rater reliability, with a resulting Cohen’s kappa of 0.97. Observations of the nine video-recorded in-person interactions between QT and AL participants were inductively coded by a single coder into 46 codes to identify the interactors’ verbal and non-verbal responses to the robot. Coding of non-verbal interactions captured gestures such as clapping, pointing, nodding and leaning towards the robot, whereas verbal interactions were coded into types of interaction such as questions, greetings, jokes and complements. The dynamics between the participant and the robot, such as whether the participant agreed or disagreed with the robot, was also coded.

## 4 Results

### 4.1 Meaning and happiness

#### 4.1.1 Survey results

**FIGURE 3:**
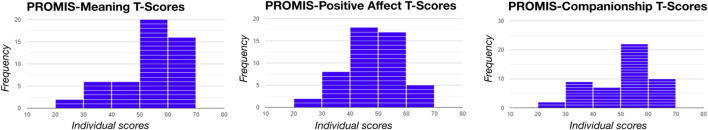
Survey participants’ PROMIS T-scores for meaning and purpose (left), positive affect (center), and companionship (right) subscales.

Results from the *Ikigai-9 scale*, *K-1 scale*, and *PROMIS Meaning and Purpose Subscale* revealed that the majority of adults in our sample had high *ikigai*, or sense of meaning and purpose. Classified according to the *K-1*, whose assessment guidelines were originally created for the Japanese population, 32 participants had high or very high *ikigai*, while five had low or very low levels. 13 participants had K-1 *ikigai* scores that were “neither high nor low.” On the *PROMIS Meaning and Purpose subscale*, 26 participants had average levels of meaning, falling within one standard deviation of the average of the general US population (scores between 40 and 60) (Figure 3 left); 16 participants scored one standard deviation above average. The average was 53.3 (SD = 11.25), indicating an average (compared to the US general population) sense of meaning and purpose. Although the *ikigai-9 scale* does not specify a determination of *ikigai* levels based on score, we do note that scores tended to be high, with 33 participants scoring between 35 and 45 (the maximum); all survey participants’ scores averaged 37.1 (SD = 5.7). For comparison, previous literature found the average *ikigai*-9 score to be 29.7 (SD = 6.3) for one sample of Japanese OAs (Seko and Hirano, 2021), while another found it to be 33.9 for a “high life purpose” group of community-dwelling OAs in Japan (Tsuzishita and Wakui, 2021).

We asked our survey participants to name between three and five sources of meaning or happiness. *Family* (*N* = 25) emerged as the number one source, such as talking to family, visiting family, or their role in the family. This was followed by *exercise* (*N* = 16), *reading* (*N* = 12), and *work* (*N* = 12). Though slightly less common, *friends* (*N* = 10), *helping others* (*N* = 10), and *performing domestic tasks* (*N* = 9), such as general home care, taking out the trash, and mowing the lawn, were also regularly mentioned. As to friends, this included performing activities with friends, such as playing music or travelling, visiting, or simply having friends. We note participants in this sample had few self-reported health problems and or physical limitations, reporting high confidence in their ability to perform activities of daily living, such as household chores and exercise.

#### 4.1.2 Online interview results

The above mentioned results were echoed in the interviews with eight individuals recruited from M-Turk, with whom we discussed *sources of happiness* and *sources of meaning*. We coded 11 sources of happiness. Family was the most common (*N* = 4), followed by work (*N* = 3) and helping others (*N* = 3), similarly to our survey data. However, unlike the survey data, three new sources of happiness emerged: *pets* (*N* = 3), *not working* (*N* = 2), and *independence* (*N* = 2). Participants typically mentioned multiple sources of happiness. Though everyone identified sources of happiness, this did not mean they were happy in a more general sense. For example, MT-P7 reported feeling no cheer (sense) in the last 7 days, even though he mentioned several activities (source) that make him happy.

When asked about meaning (what brings people a sense or value or worth, or a reason for living), people provided fewer sources, though it is possible that this was because the question was asked much later in the interview. Helping others was the most common response (*N* = 3), followed by *relationships* (*N* = 2). In one participant’s (MT-P4) words, *“Just being able to be helpful to people. Being able to be there for people who need you when they need you. I think that’s what makes life worth living.” Not knowing or having meaning* in their life was reported by two participants in our small sample.

#### 4.1.3 In-person interview results

When asked about sources of meaning in life or sources of happiness, participants from the assisted living (AL) facility most commonly mentioned *relationships* (*n* = 12), such as being around children and grandchildren, and helping others (*n* = 8) as bringing meaning to life, and *family* (*n* = 27) and *friends* (*n* = 18) and *helping others* (*n* = 5) as sources of happiness. *“[Spouse] and I think about our boys and myself. And so I have purpose, you know, I don’t feel lost in the wilderness”* (AL-P2). *Helping others*, often meant cooking for family, babysitting grandchildren, helping neighbors or talking to someone about their problems. Family and helping family members seemed to be the sole ‘reason for living’ for some who seemed otherwise unhappy with their life—*“Well, I have obligations to other people (family), I can’t let them down (cannot give up/die yet)”* (AL-P8).

For others, happiness was associated with doing favorite activities, like painting, sewing, or cooking. Some mentioned religion (*n* = 2) or past pets (*n* = 2) as sources of happiness.

Participants reported unhappiness due to deteriorating health and their transition to AL, away from family and friends. Many participants (*p* = 5) had negative perceptions of themselves, *“I really don’t like myself a whole lot … I have a lot of [health] trouble … That makes me most of the time quite unhappy.”* (AL-P8). Several participants (*N* = 7) felt they were not useful to their family or friends anymore. Getting help from others led to feeling like a burden; e.g., *“Seeing friends makes me happy. But I don’t like to call them because then they have to do something to pick me up or do something like that.”* (AL-P7). Despite the barriers, participants (*N* = 5) looked for meaning in taking care of themselves and carrying out everyday activities.

When asked about their feelings, all nine AL participants mentioned feelings of contentment (*n* = 40). Participants seemed to have found ways to overlook unhappiness posed by their deteriorating health and find meaning and happiness in day to day interactions and doing activities structured for them by the AL facility. Participants discussed how they required no significant reason to be happy (*N* = 5). *“My reason for living, is … I try to enjoy things that come; So I’m really happy with my life but I can’t think of any way to make it better.”* AL-P3.

### 4.2 Social connectedness

#### 4.2.1 Survey results

Most OAs in our survey had average levels of companionship and emotional support; on the PROMIS Companionship scale, 11 of the 50 participants scored at least one standard deviation below the US average, and 10 above it (Figure 3 right). Individuals rated their emotional support even higher according to PROMIS Emotional Support scale scores, with 15 participants scoring one standard deviation above the US population average, and only eight below. We also collected data on how often people interacted with others. Participants met with close family and friends about twice a week and socialized with neighbors about as often. However, most did not participate in any group or organized activities, or volunteer.

#### 4.2.2 Online interview results

Independently living OAs mostly connected to their social network by phone (*N* = 6), in-person (*N* = 5), or through video software, such as Zoom (*N* = 4)^4^. Participants had two main sources of social connection: family (*N* = 8) and friends (*N* = 6). The degree to which they were connected differed, and many participants mentioned having overall few social connections (*N* = 4).

Those reporting few social connections were not necessarily less happy than their counterparts with a broader social network; they often discussed how this was how they had designed their life or what they’d become accustomed to: *“I think at this point, being alone, I’m starting a whole new kind of creative process in my life. So I’m actually happiest right now being alone.”* (MT-P3) There were, however, some moments in which these individuals seemed to feel isolated, even describing strategies such as keeping the TV on to make themselves feel less lonely: *“The other thing that’s going on in the background [is the TV],…that’s my other companion … so I don’t feel totally isolated.”* (MT-P3).

### 4.2.3 In person interview results

Like the independently living OAs, the AL participants mentioned that their social connection was mainly with family (*N* = 9) and friends (*N* = 6). These social connections provided companionship (*N* = 7) and happiness in their lives: *“We (family) like to walk and talk with each other. I think we’ve made a considerable bond between us, and that makes me really, really happy”* (AL-P3). Although they were often far from their family and friends, the existence of these connections and participants’ memories of them made them content and kept them from being lonely.

Social connections provided participants with company for doing enjoyable activities (*n* = 10); sharing everyday experiences (*n* = 5); sharing their deepest issues and problems (*n* = 9); and companionship (*n* = 8). Being in an AL facility, participants felt they had companionship at all times—*“There are always people (other residents) to talk to here, you know? They are generally very intelligent. Fun to know a couple of them.”* (AL-P4). However, when participants told us with whom they would have discussions related to their everyday experiences and problems, they often mentioned their family or close friends, as opposed to the other residents. One resident commented on a lack of social opportunities conducive to deep conversations and sharing personal experiences. *“When I have the opportunity, I do what I do, but I can’t create the opportunity (to have deep conversations). Someone else has to do it. So I’m very grateful that somebody has arranged this meeting (the research interview)…. That would be very helpful for both of us. Because someone like me, can’t do it on my own.”* (AL-P9).

### 4.3 Influence of living situation on independently living OAs

Whether participants lived alone or with others affected their reported sources of meaning and happiness in interviews and surveys. Though survey results showed ratings of meaning and happiness were similar between groups, their sources varied (Table 2).

**TABLE 2:** Summary of findings (sources of ikigai).

Participants	Meaning	Happiness	Social connection	Barriers
Lives Alone^4,23^	Inherent in life, activities of daily life, uncertain	Independence, not working, (solo) activities (e.g., exercise, learning, reading, painting, watching TV), family, pets	Few social connections (not seen as a negative)	Lack of structure/organization, procrastination, knowing what to do, location
Lives with Others^4,27^	Family, friends, helping others	Family, helping others, work, activities (e.g., exercise, reading, domestic tasks, travelling, volunteering)	Family, friends	Few mentioned^a^
AL Facility^9,0^	Family, helping others	Family, friends, helping others, activities (e.g., painting, sewing, cooking)	Family, friends	Health, negative perceptions of self, feeling like a burden

In the participants’ cells, the first superscripted number is the # of interview participants; the second in the # of survey participants.

^a^ Participants (across all groups) mentioned the COVID-19, pandemic as a barrier to social interaction and engaging in typical activities.

Online survey participants who lived with others (*N* = 27) reported their top sources of meaning and happiness as family (*N* = 18), friends (*N* = 9), exercise (*N* = 9), work (*N* = 8), performance of domestic tasks such as cooking and cleaning (*N* = 7), helping others (*N* = 6), and reading (*N* = 6). These results were echoed in the online interviews of the eight individuals recruited from M-Turk. The four individuals who lived with others mentioned family most often as a source of happiness (*N* = 3), followed by helping others (*N* = 2), and work (*N* = 2). Helping others and important relationships were the most common sources of meaning. In the words of one participant (MT-P8): *“My husband … Absolutely. My friends. Absolutely. They make life really worth living. And relationships really … Or just helping somebody do something or find some thing you know.”*

In contrast, online survey results for the 23 participants living alone revealed top sources were family (*N* = 7), exercise (*N* = 7), reading (*N* = 6), learning (*N* = 5), and memories of past events (*N* = 5). Therefore, while family, exercise, and reading were important to both groups, differences in friends, helping others, performance of domestic tasks, learning, and memories emerged. Additionally, although family was a top source for both groups, it was mentioned more frequently among those who lived with others than those who lived alone (67% vs. 30%).

Additionally, for those who lived alone, interview results showed their main sources of happiness related to maintaining their independence (*N* = 2) and not working (*N* = 2). Not working related to maintenance of their independence, and allowed them to pursue activities they cared about. As one participant pointed out (MT-P5): *“The job that I had last was very involved, so I didn’t get to do much of anything. Now, [I’m] working on those things that I missed … That’s why, so I said, what makes me happy, it’s not working. And it’s amazing.”* They mentioned engaging in activities far more often, and had a more diverse set of pursuits, than their counterparts. These were more likely to be “solo” activities (learning, drawing, painting) rather than the group activities (outings, volunteering, talking to others) more common for those who lived with others.

Family (*N* = 2) and pets (*N* = 2) were also sources of happiness for individuals who lived alone. They all also reported a desire for continued learning (*N* = 4), which supports our survey results. A desire for change (*N* = 4) was also pervasive, and this mainly had to do with resolving barriers to their happiness (i.e., moving to a larger city, changing jobs or retiring). As for meaning, the individuals who lived alone either had trouble defining their meaning in life (*N* = 2), or found it to be inherent in life itself or the small actions they took in the course of their lives (*N* = 2). One participant (MT-P1) explained: *“It’s not one or two things. It’s everything. It’s just life is meaningful.”*

Those who lived alone also talked about structural barriers to achieving or maintaining their purpose. Specifically, they talked about not being efficient in organizing their days or procrastination in getting their preferred projects done (*N* = 3) or difficulty in knowing what their next step or change in life should be (*N* = 2). This latter concern seemed to be related to whether they recently retired or were thinking about it. *“So I might, maybe I could sell that rather than working, like actually start to make money from my art again. I thought about horseback riding but that was just like, if I fall off I’m getting a little too old to be falling off horses. So like what? I don’t know, you know?”* (MT-P3). They also talked about location as an additional barrier to social connection, if they lived in a rural or suburban area without easy access to people (*N* = 2).

All four people interviewed online who lived alone reported few social connections, while all four individuals who lived with others reported larger social networks. However, as reported in the social connectedness Section 4.2, having few social connections was not typically negatively viewed and in fact was often seen to contribute to the richness of their lives. Participants were therefore somewhat ambivalent about seeking new friendships. For example, MT-P3 noted that if she could find new friends that shared a similar mindset as her she would, but she preferred to be alone than with the people around her with whom she felt she did not relate.

### 4.4 Robot perception

**FIGURE 4:**
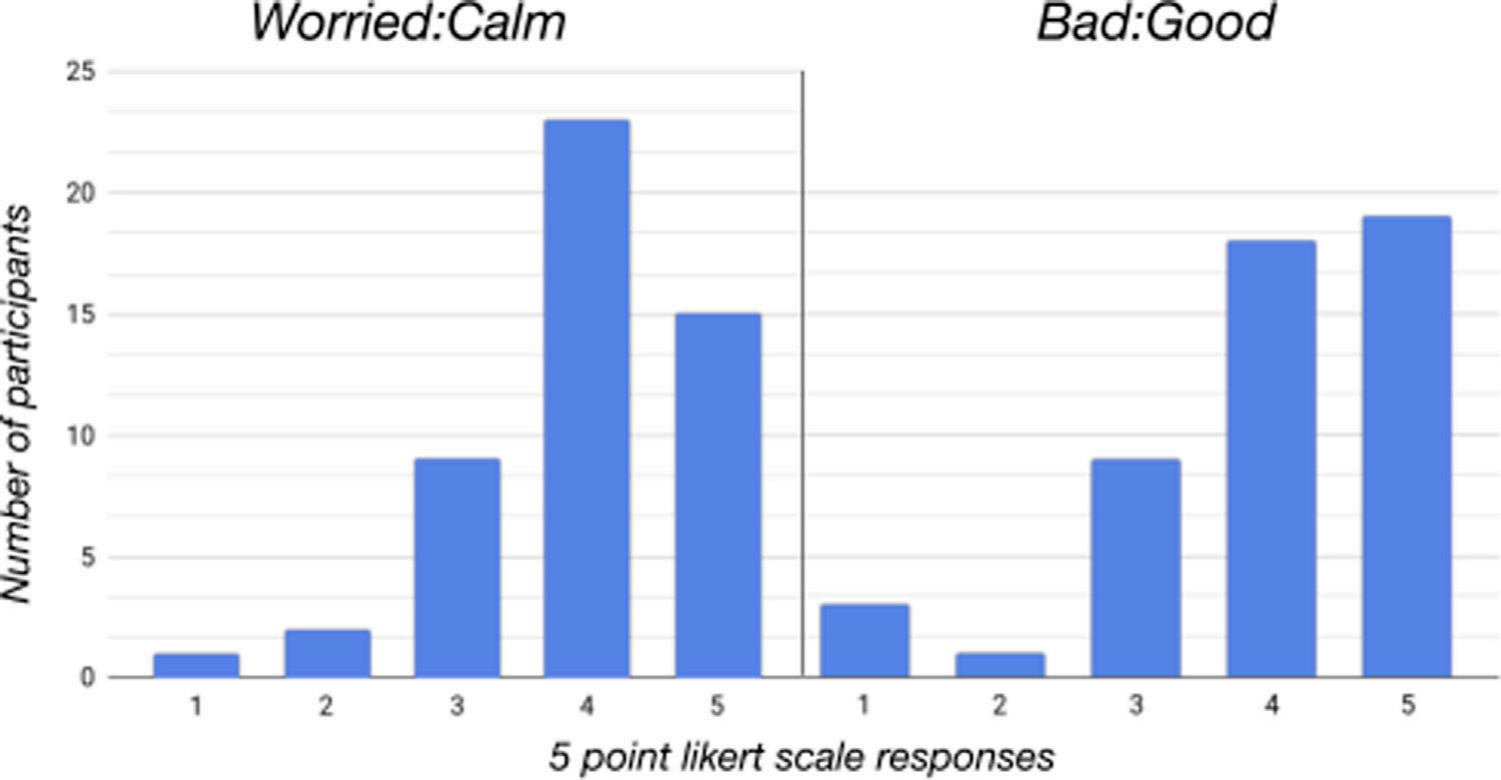
Feelings towards having the QT robot in one’s home (based on video stimuli).

The **Robot Perception survey** found that the overall perception of QT as presented on video (Figure 4) and through **live interactions** produced similar results. In both methods of presentation, QT was shown performing ikigai and well-being related behaviors. Survey results showed the robot’s perceived usefulness, trust (whether participants would trust and follow advice the robot gave), and perceived sociability (participant’s desire to interact and converse with the robot) were highly rated (Table 3). Both online survey participants and in-person (AL) participants expressed being comfortable with the idea of talking to QT about their life experiences, memories, strengths, past, and future plans, though survey participants were more comfortable talking about positive memories and experiences than negative ones. 18 people indicated they were somewhat or extremely uncomfortable talking about bad memories, while only five people noted the same for good memories.

**TABLE 3:** Almere survey results.

Almere subscale	Mean	Std. dev
Anxiety	2.35	1.10
Attitudes toward technology	3.87	1.03
Facilitating conditions	3.93	0.71
Intention to use	3.77	1.03
Perceived adaptability	3.79	0.69
Perceived enjoyment	3.78	1.01
Perceived ease of use	3.98	0.62
Perceived sociability	3.77	1.00
Perceived usefulness	3.96	0.98
Social influence	3.53	0.86
Social presence	3.06	0.94
Trust	3.44	0.85

Based on open-ended responses on desired uses of QT, top requests were for exercise (*N* = 23), domestic tasks such as cooking and cleaning (*N* = 21), as an informational assistant to schedule appointments, set reminders, and report the weather and news (*N* = 21), and for game play (*N* = 20). Use as a conversational partner (*N* = 15) and for learning (particularly language learning) (*N* = 14) were also common.

In person (AL) participants were even more open to conversations with QT (*N* = 9) and imagined those conversations to be playful and entertaining. They were particularly interested in using the robot to learn a new skill (*N* = 8), learn about technology (*N* = 9), and showed much interest in playing games with it (*N* = 6). While they were open to the idea of a robot motivating them for exercise (*N* = 9), most (*N* = 8) thought a robot could not give them suggestions on healthy diet and lifestyle and they would rather have their companion staff at the AL facility guide them. *“Does the robot know more about the food? There are people who are already telling me more about the food … it is not useful for me.”* (AL- P4). They were positive about having a robot telling them the weather or current news (*N* = 8), as many were familiar with smart home assistants with similar functionality; however, they thought the support staff at AL facility were a better choice for assisting them with scheduling and medication reminders (*N* = 6).

### 4.5 Behavioral analysis of live interactions with QT

Participants (*N* = 9) from the AL facility interacted one-on-one with QT for a total of 53 min, which we video recorded. We used these videos to analyze participants’ behaviors toward the robot.

Participants addressed QT as if it were a social being—“I am talking to one of the brightest littlest persons I know” and even called it a friend at times- *“Oh but you are [already] my friend. Yes.[…] I would take good care of you.”* They used affectionate words while interacting with QT (*n* = 30), complimenting it—*“oh my.. what a smile”* or *“You are very cute”* and gave names to QT. Sometimes they showed their affection explicitly, through statements such as *“I like you”* and *“I love my family and they would love you!.”*

Overall, participants seemed comfortable engaging in conversation and activities with QT, and often engaged verbally and non-verbally with the robot. Participants paid close attention to QT’s actions (*n* = 265) and had their gaze fixed on QT most of the time. They smiled (*n* = 101) in response to QT’s actions, made gestures such as “thumbs up” to acknowledge and agree, and “clapped” (*n* = 3) to express their enjoyment. They joked (n = 11) when QT said -“I hope you will take good care of me,” such as by saying—*“We’ll have to!”* AL-P6.

Participants often imitated or mirrored QT’s facial expressions, movements, and body positioning. Of the 70 instances coded, only 25% occurred when the robot asked participants to follow it, such as in the exercise task (*n* = 18). 75% occurrences were unprompted (*n* = 52) and happened throughout the interaction. Participants also imitated QT’s facial expressions such as blinking and smiling (*n* = 79). Besides imitation of gestures and expressions, participants repeated what QT said (*n* = 14).

Participants often responded to QT’s actions and questions (*n* = 26). In response to QT’s requests for certain actions on their part, participants often agreed (*n* = 27). Some even agreed to take care of QT: *“Oh, I will take good care of you, absolutely.”* AL-P5 Likewise, when QT suggested some activity for the day, they accepted the suggestion saying, *“Oh, thank you!”* .AL-P3 Upon QT’s suggestion to go for a walk one participant said (smiling) *“Oh ok, Maybe I will take a little walk with you. Okay. Alright I will do that thank you.”*

Several participants (*n* = 32) shared something about themselves (*n* = 19), their plans for the day (*n* = 6), or something about their family and friends (*n* = 7) with QT. When QT introduced itself, participants often introduced themselves in return (*n* = 4). Likewise, they willingly shared their plans for the day and information about their families: *“I have three children [..] they would love you! I am gonna tell them about you.”* AL-P8.

### 4.6 Influence of living situation on robot perception

Just as sources of meaning, happiness, and social connection differed based on whether participants lived alone or with others, so too did their views of the *ikigai* robot. Perception of QT was overall more positive for those who lived with others than those who lived alone, with more of the former group choosing they felt “good” and “positive” about having the robot in their home (at 84% vs. 69%, and 83% vs. 58% for each adjective, based on selecting a four or five on our Likert-scale); (Figure 4). Likewise, ideal ways of engaging with the robot also differed. For those who lived with others (*N* = 24), participants reported wanting to use the robot for exercise (*N* = 16), either to instruct them in exercises or to remind them to exercise; to engage in play or games (*N* = 12); act as a conversational partner (*N* = 10); and as an informational assistant (*N* = 8), in order to help schedule appointments, set reminders, and to give them weather and news information. However, participants who lived alone (*N* = 26) wanted to use the robot for domestic tasks such as cooking and cleaning (*N* = 14), as an informational assistant (*N* = 13), and for games/play (*N* = 8).

When asked if they felt the robot was too intrusive, most people who lived with others disagreed, with 19 of these 24 participants indicating they disagreed or strongly disagreed. As well, they liked that QT gave suggestions (*N* = 20). On the contrary, most participants who lived alone (*N* = 26) found QT too intrusive (*N* = 16), agreeing or strongly agreeing when asked this question. While the majority were positive about the robot giving suggestions (*N* = 16), less participants living alone indicated being so than those who lived with others.

## 5 Measuring meaning, EWB, and ikigai

One of our goals in including several different survey measures relating to meaning and purpose, developed in Japan and the US, was to see how these measures compared with each other and which may be more suitable for future use.

Comparing the K-1 scale developed in Japan to the PROMIS meaning and purpose subscale, the latter of which was validated with a US population, we find that more participants are classified as having high *ikigai* (meaning) based on the K-1 scale. However, the distributions of the K-1 and the PROMIS scores were similar, indicating they may be capturing the same concept (and this may be slightly different than the *ikigai*-9), even though the interpretation of these scores differed. In fact, we find that the K-1 and PROMIS (raw scores) were strongly and significantly correlated (r(48) = 0.79, *p* = < 0.001). Although the combinations of PROMIS and *ikigai*-9, and K-1 and *ikigai*-9, were also correlated, they showed weaker relationships (*r* = 0.67 and 0.69, respectively). The stronger overlap between the K-1 and PROMIS may be somewhat expected, given that some items on the *ikigai*-9 relate to happiness and activities, reflecting a different conceptual understanding of ikigai (as both a eudaimonic and hedonic phenomenon). Our findings, and previous literature, indicate that happiness and meaning are separate conceptually; the concept of happiness (*shiawase* in Japanese) has been connected with hedonic well-being, while *ikigai* is related to eudaimonic well-being (Kumano, 2018). However, several ikigai-9 questions pertain to happiness and interests (e.g., “I often feel that I am happy.” and “I am interested in many things.”). This likely reflects an understanding by certain researchers that *ikigai* has both a eudaimonic and hedonic component. This may also be reflective of a cultural difference, and it is possible that in Japan, where both the ikigai-9 and K-1 were developed, happiness and meaning have a stronger correlation, whereas the focus of our study was the US population. The K-1 and PROMIS both focus more directly on meaning, fulfillment and, in the case of the K-1, family and perceived contributions (which we find to be an important source of many OAs’ *ikigai*).

To measure well-being holistically, Huta (2013) recommends four aspects of well-being be captured: positive affect, negative affect, life satisfaction, and meaning (Huta, 2013). Our work is important in defining how meaning may be measured within HRI, in addition to or separate from the three hedonic factors, as we tested several potential scales. Specifically, to assess meaning for the US population and cross-cultural research, we recommend use of the PROMIS Meaning and Purpose scale or the K-1 scale, rather than the ikigai-9 scale.

## 6 Comparing meaning, social support, and happiness

Our findings indicate that, while related, a sense of meaning is not contingent on a wide social support network. This can be confirmed by the only moderate correlation between the PROMIS Meaning and Purpose scale and the PROMIS Companionship scale [r(48) = 0.39, *p* = 0.006], though there was a stronger correlation between the PROMIS Meaning and Purpose scale and the PROMIS Emotional Support scale [r(48) = 0.61, *p* < 0.001]. However, our survey and interview findings show companionship is *more important* for OAs who live with others than for those who live alone. Supporting this point, based on the relevant PROMIS scales, we find no relationship between meaning and companionship for those who live alone [r(21) = 0.24, *p* = 0.275], and a large significant relationship for those who live with others [r(25) = 0.5, *p* = 0.007]. Emotional support, on the other hand, showed the same strong, positive association to meaning regardless of living situation. This indicates that further connecting OAs who live with others with family and friends is likely to foster their sense of meaning and purpose, but **other means of increasing meaning, or a focus on fostering a few deep connections if lacking, may work better for those who live alone**.

Additionally, meaning and happiness were strongly correlated when comparing the PROMIS Meaning and Purpose scale and the PROMIS Positive Affect scale (r(48) = 0.75, *p* < 0.001); (Figure 3 left and center). This is in line with previous research showing a strong correlation between the two concepts (Heintzelman, 2018). However, this correlation was weaker when we evaluated just those who lived alone [r(21) = 0.59, *p* = 0.003] compared to those who lived with others [r(25) = 0.84, *p* < 0.001]. While this shows that meaning and happiness relate, it illustrates that there are different experiences and mindsets that may contribute to one but not the other. This is especially true for those who live alone, whose main *sources of happiness* (related more heavily to their independence and solitary activities than those who lived with others) differed from their main *sources of meaning* (which were often viewed as inherent in life and activities of daily life, missing, or difficult to define). The fact that family and helping others were integral to both the happiness and meaning of OAs who lived with others explains the very high correlation between these scales for this group.

## 7 Designing an ikigai robot

### 7.1 Designing for ikigai vs happiness and social connection

Designing robots for *ikigai* (meaning) has several commonalities with designing assistive robots for increasing happiness and social connectedness. In fact, *ikigai* often (though not always) relies on being socially connected to others. As well, there is overlap for some individuals in that which makes them happy and that which provides them meaning. However, there are also notable differences, in both specifics of implementation and scope.

First, though happiness and meaning often co-occurred, we found that in our data, as well as in the well-being literature more broadly, there are participants who experience the hedonic sense of well-being without EWB. There were also participants who experienced EWB without hedonia (happiness). However, those who experience both senses of well-being (hedonic + EWB) have greater well-being and a more “balanced” sense of well-being than those who only experience one (Huta, 2017). Therefore, it is important to *go beyond* thinking about well-being strictly as a state of happiness, which is typically the case in HRI literature. For robots to be implemented in this manner, they would need to assess an individual’s sources and felt sense of *ikigai*, likely through speech or assisted text-based interactions. This may be highly personal to each individual. As changes in meaning in life take longer to occur than changes in happiness, this would need to be re-assessed on a longer-term basis.

We also find that although individuals had varied sources of happiness, they reported fewer sources of meaning. This suggests that there are a limited number of areas to focus on for each individual when helping to establish or improve meaning in life, though there may be a wider array of experiences to increase happiness. Activities specifically seem to be associated with happiness, but not meaning, for many OAs. Therefore, increasing meaning may require a more targeted approach and an ability for the robot to learn which people or experiences are associated with meaning for each individual.

In fact, for robots exclusively meant to increase happiness for those who live alone, we might focus more on activities commonly associated with first person *ikigai* (personal sources), namely activities such as hobbies, exercise, and learning pursuits. However, these activities were not always associated with meaning for them. For example, two of the four individuals interviewed who lived alone talked about not knowing or having meaning, even though they were engaged in a multitude of activities. This suggests that: a) additional sources of meaning (such as volunteering or connecting with a few family members or friends in a deep, emotionally supportive way) may positively influence their meaning, even if does not have a profound affect on their happiness, and/or b) although they have a sufficient *source(s)* of meaning, they are lacking a *sense* of meaning. A robot could therefore help foster this sense, for instance, by daily reminders for reflection on certain aspects of one’s life. Likewise those who lived with others (including at the AL facility), primarily discussed obtaining a sense of meaning (and happiness) at the second person level of ikigai (interpersonal sources), such as connections with their family. However, the ikigai model suggests that they are likely to experience an increased sense of meaning by also having an activity (first person) that is meaningful to them, along with an opportunity to effectively give back to the community (third person level of ikigai). In this case, the robot could make appropriate suggestions (after learning about the individual) which could fuel self-development and/or community-level contribution.

Though being connected with others is an important aspect of both meaning and social connection (and happiness), the *who* and *how* of social connection differs when designing for *ikigai*. Regarding the who: a robot meant to boost ikigai should not be designed to spur just any interaction, but specifically to **enhance family bonds**. This relates to the second person level of *ikigai* (interpersonal source). Participants’ live interactions with QT suggested they are willing to share their personal experiences, plans for the day, and information on family and friends without much reservation. As such, robots may hold potential to learn about and initiate family connections. Regarding the how: robots used to create social connection are indiscriminate to the directionality or hierarchy of these connections. However, our research suggests that many OAs may be hesitant to reach out to people that they know, for fear of feeling like a burden (if they need something or if they feel like they are reaching out too often). It also shows that OAs derive a sense of meaning by helping others. Therefore, social robots for ikigai should encourage older adults to connect by finding small (or large) ways to **do things for other people**, so that they themselves feel useful.

Perhaps most special to robots created to foster *ikigai* is this focus on helping OAs find more opportunities to help others. Most individuals studied, whether living independently or in assisted living, indicated that they did not engage in group or organized activities, or volunteer. Some OAs also mentioned that they did not know of or were not comfortable initiating these activities themselves. Therefore, this third person level of ikigai (community) was missing as a source of meaning. This void may be filled through QT suggesting **volunteer opportunities** or that individuals simply check in on a neighbor to see if they need anything. This also aligns with *ikigai’s* unique sense of social participation that focuses on doing for others, instead of just being with others, with the latter often being the focus of HRI work designed to generally increase social connection among older adults. An emphasis on doing things for others is also likely to make OAs more likely to engage, as they often prevented themselves from reaching out to others when they perceived they would burden them.

Furthermore, taken together, our results suggest that ikigai maintenance may be important for some OAs, while ikigai source and sense generation is necessary for others. *Ikigai* maintenance may be comprised of **reminders to engage** in the experiences that already serve as OAs sources of *ikigai*. This requires an intimate knowledge of OAs’ sources of *ikigai* and an ability to personalize the robot to each OA. Increasing *ikigai*, on the other hand, may be comprised of **suggesting new experiences** and potential sources of *ikigai*. This requires an intimate knowledge of the OAs’ preferences, abilities, physical limitations, family, living situation, and context-of-use, along with community activities and possible volunteer opportunities. From our live interactions, we saw that activity suggestions provided by the robot may receive positive responses and compliance from interactors. Additionally, increasing *ikigai* may simply require reflection on one’s existing life experiences and sources of *ikigai*, to help increase its felt sense. This may take the form of the robot **prompting daily reflection**, such as what was showcased in the video shown to participants. It may also take the form of encouraging OAs to **share their positive memories and experiences** with the robot, which nearly all OAs indicated being willing to do.

Additionally, though our research is focused on EWB, specifically *ikigai*, it also acknowledges that *well-being is best designed for holistically, as a combination of EWB and happiness*. For example, we found that many participants indicated wanting to use the robot to help them exercise. Exercise is positively correlated with happiness (Khazaee-Pool et al., 2015; Zhang and Chen, 2019). Live interactions with QT showed how it could motivate interactors to exercise as well as prompt behavioral changes, as participants imitated the robot’s body movements as well as facial expressions. Cognitive and skill development assistance from the robot was a feature participants imagined having, especially for language learning, and this capability was a source of happiness, especially among those who live alone. As well, although game play and humor are also not associated directly with *ikigai*, we saw during OAs’ interactions with the robot that these would likely help OAs build rapport and maintain engagement with the robot. Therefore, though not directly related to meaning, this additional functionality helps increase well-being more holistically and also helps keep engagement with the robot high, so individuals are also more likely to use the robot for ikigai-specific purposes.

### 7.2 Designing for diverse OAs

We discuss general guidelines for designing a robot to support OAs’ *ikigai* above, but also note that certain characteristics of the OA user should frame implementation. For those who are independently living with others, a *“socially enabling”* robot designed to engage and help them to connect socially, as well as to encourage them to help others (friends, family, the community), should be at the forefront. These OAs also specified they would like the robot to talk with them, and to be proactive in its suggestions. They did not think the robot would be intrusive to their home, perhaps because they are used to sharing it with others (e.g., their spouse). Our current implementation of QT, as shown in the stimulus video, aligns well with this population’s desires. As many of the individuals in our study already had a source and a sense of *ikigai*, a robot may serve to facilitate and help them maintain this sense.

For OAs living alone, the robot should help them structure their day to optimize their time and maximize doing the things they love. The robot should be a *functional tool* to enhance their ability to maintain their independence. It should also help them maintain their cognitive heath, and make it easy for them to participate in the activities they love (like learning). It should further help them explore what their purpose might be if it is lacking, which may be especially relevant post-retirement. For these individuals, the robot itself could be less social so as to seem less intrusive to their space. Our current design of QT may not be appropriate for many OAs who live alone.

It is also important to be mindful of not having a robot take over tasks that bring *ikigai*, happiness, or fulfillment to people’s lives. For example, OAs who lived with others often indicated performance of domestic tasks (cooking, cleaning, etc.,) as a source of happiness, and this was not something they suggested as a task for the robot. On the contrary, those who lived alone did not specify this as a source of happiness, and it was one of the top requested tasks for the robot.

Similar to OAs independently living with others, those in organizational settings, such as in assisted living, could benefit from a robot designed to engage with them, help them connect with family and friends, and encourage them to help others within and outside their facility. Since AL participants thought of social conversations as a meaningful contribution, providing a way for them to help others could be as simple as creating opportunities for them to have deep and meaningful conversations with other community members.

Additionally, it seemed happiness for these OAs came from having structured activities throughout the day. Since these participants most often had one or two hobbies and activities that brought meaning to their lives, in order to suggest activities for the day, the robot would need to know about their past pursuits of these hobbies and how they created meaningful experiences to them. Considering their health and mobility limitations, the robot would then need to find and suggest alternative ways to provide them with similar experiences.

While we outline several recommendations above based on living situation, we note that there are likely to be other characteristics that influence the diversity of OA experiences and preferences. For example, it is worth considering whether group differences exist based on cultural background or exact age bracket. As well, though we found no apparent differences in survey responses based on gender, we did not explore differences in their interaction with, or expectations of, the robot, and this may also be worth exploring in future work. Indeed, past research suggests that gender-relevant differences exist both in the experience of ikigai (Shirai et al., 2006; Park, 2015) as well as interactions with, and expectations of, robots (Broadbent et al., 2009). However, we believe this work presents an important step in designing for heterogenous OA experiences.

## 8 Conclusion and future work

Our larger project focuses on creating robots to foster OAs’ sense of purpose and meaning in a personalized way, and our study is the first step in developing a grounded understanding of how that can be accomplished for OAs in the United States. We believe that this focus is grounded in the fact that there are many positive aspects of aging and OAs can engage with their social network and community in ways that allow them to share their knowledge and contribute, instead of aging being treated as a disability. We highlight differences in design recommendations for a robot meant to increase ikigai versus one designed to focus on social support and social connection—including suggestions for volunteering, suggestions to engage in new hobbies and activities, promoting daily reflection, and a focus on building interpersonal connections specifically by encouraging OAs to help other people, instead of merely being with them. Our findings show the need for understanding OAs’ specific living situations, as each group (those who live with others, those who live alone, and those who live in assistive living facilities) expressed distinct preferences towards an ikigai robot. Furthermore, each person’s sense of purpose and meaning is dynamic and evolving throughout their life. It therefore is necessary for our robot to learn about users’ preferences and to change with them, in order to respond to them as dynamic individuals.

## Data Availability

The raw data supporting the conclusion of this article will be made available by the authors, without undue reservation.

## Footnotes

1
https://luxai.com/humanoid-social-robot-for-research-and-teaching/#hardware-specification.

2 Referenced as MT-P# for those on M-Turk and AL-P# for those in Assisted Living.

3 N refers to number of participants and n refers to number of instances.

4 This research took place during the COVID-19 pandemic. This influenced the frequency and methods of social engagement for many of our independently living OAs; thus results should be interpreted with this caveat in mind.
